# Distinguishing Features of AAA-Healthcare Partnerships: Business Acumen Is Necessary but Not Sufficient

**DOI:** 10.1093/geroni/igab046.1928

**Published:** 2021-12-17

**Authors:** Leslie Curry, Emily Cherlin, Adeola Ayedun, Chris Rubeo, Traci Wilson, Amanda Brewster, Jane Straker

**Affiliations:** 1 Yale School of Public Health, New Haven, Connecticut, United States; 2 Yale School of Public Health, Yale Global Health Leadership Initiative, New Haven, Connecticut, United States; 3 University of California, Berkeley School of Public Health, Berkeley, California, United States; 4 USAging, Washington, District of Columbia, United States; 5 Miami University, Oxford, Ohio, United States

## Abstract

Stronger relationships among service providers in the health care and social service sectors may contribute to positive outcomes such as lower health care use and spending. Such partnerships have grown in recent years, including Area Agencies on Aging (AAAs) contracting with health care organizations, and their impact on health care utilization has been demonstrated. Nevertheless, knowledge about how AAAs establish and manage successful collaborations is limited. This study was designed to understand how AAAs in regions with low levels of avoidable health care utilization develop and sustain partnerships with health care organizations. We conducted an explanatory sequential mixed-methods, positive deviance study. In the quantitative phase, we identified 8 AAAs with multiple health care partners serving areas with little utilization of nursing homes by residents with low-care needs, and 3 with few partners and high utilization for comparison. In the qualitative phase, we identified key informants within AAAs and their partners for in-depth interviews (total n = 123). We used the constant comparative method of analysis to identify 5 factors that characterized partnerships in the highly-partnered, low-utilization sites: 1) Regional context (e.g., breadth of health care provider market, cross-sectoral coalitions), 2) AAA human resource assets (e.g., community expertise, business acumen), 3) AAA organizational culture (e.g., visionary leadership, risk taking), 4) Interdependence among organizations (e.g., mutual benefit, alignment), and 5) Interpersonal dynamics (e.g., trust, relationships). The importance of these regional, organizational, and relational factors suggests that AAA business acumen is necessary but not sufficient to build and sustain robust cross-sectoral partnerships.

